# The Dynamics of Cell-to-Cell Water Transport and the Involvement of Aquaporins in Response to Apoplast Blockage in the Roots of Intact Maize Plants

**DOI:** 10.3390/cells14120902

**Published:** 2025-06-14

**Authors:** Maksim Suslov

**Affiliations:** Kazan Institute of Biochemistry and Biophysics, FRC Kazan Scientific Center, Russian Academy of Sciences, P.O. Box 30, 420111 Kazan, Russia; makscom87@mail.ru

**Keywords:** water transport, cell water permeability, cell-to-cell pathway, aquaporins, apoplastic pathway, plant roots, spin-echo NMR, transpiration

## Abstract

Investigating the contribution and interaction of water transport pathways in plant roots is important for understanding the functioning of the root hydraulic system. In this study, the real-time dynamics of lateral water transport along the cell-to-cell pathway and the diffusional water permeability of cells in the root suction zone of whole maize plants were investigated non-invasively by spin-echo NMR in response to rapid blockage of root apoplast. Apoplast blockage was carried out by insoluble precipitates using an original approach based on alternate incubation of whole plant roots in aqueous solutions of K_4_[Fe(CN)_6_] and CuSO_4_. In the first stage after the apoplast blockage, the water transport along the cell-to-cell pathway and the diffusional water permeability of root cells was decreased 2.5 times. Using inhibitory analysis and gene expression analysis, it was shown that root aquaporins are involved in the decrease in cell-to-cell water transport in response to apoplast blockage. After an initial decrease, the cell-to-cell water transport was restored to initial values. At the same time, there was a partial compensation of the transpiration loss caused by the apoplast blockage. It is assumed that the apoplastic water flow in plant roots can modulate the cell-to-cell water transport and functional activity of aquaporins.

## 1. Introduction

Water transport in plants is one of the most important regulated processes that ensure plant growth and development. Depending on the external conditions, the regulation of water transport in plants can occur to varying degrees in both the above- and belowground parts of the plant [1,2]. However, the root system is the most important component of the whole plant hydraulic system, providing water uptake and regulating the water status of the plant, especially under water stress. Root hydraulic conductivity is an important parameter that reflects the ability of plant roots to absorb and transport water [3,4]. According to the composite model of radial water transport, the hydraulic conductivity of roots is regulated by three different pathways of water flow: apoplastic flow along the cell walls, transcellular movement across cell membranes and symplastic movement of water via plasmodesmata [5,6]. Due to the methodological difficulties of separation, the latter two pathways are often combined into a single cell-to-cell pathway. The composite model of water transport assumes that the contribution of different water pathways to the total water transport can vary, providing a useful mechanism by which plants can respond to changing environmental conditions and stresses [7,8,9]. One of the important and challenging tasks on which researchers in this field have focused their efforts is to quantify the contribution of apoplastic, symplastic and transcellular pathways to the total water transport in plants under normal and stress conditions [10]. When conducting biological experiments and interpreting their results, as well as when constructing mathematical models of radial water transport in roots, the apoplastic and cell-to-cell pathways are often considered to be parallel and independent. However, this separation is conditional and simplifies the solution to the problems described above. In reality, the apoplastic and cell-to-cell pathways of water transport overlap. This is due to the fact that water molecules moving along the cell-to-cell route, crossing the membrane, inevitably enter the extracellular space where they are affected by the apoplastic water flux. According to some data, the apoplastic pathway, which has a volume fraction in the root of only 5–7%, can provide up to 50% of the total water flow in roots [10]. This is achieved as a result of the high rate of water flow through the apoplast, which under normal conditions is several times higher than the rate of water flow through the cell-to-cell pathway. In this respect, the rate of water flow through the apoplast and the state of water in the apoplast should, from a physical point of view, influence the time and place where water molecules will be after leaving the cell into the apoplast during the transcellular transition, as well as the number of transcellular transitions per unit time and, consequently, the magnitude of the transcellular water flow. Assuming that the rate of the apoplastic water flux can impact cell-to-cell water transport to maintain the optimal water status of cells; for example, when transpiration is altered, one would also expect changes in the water permeability of cell membranes. The contribution of the apoplastic pathway to the total water transport in the roots of some plants is known to decrease under conditions of water deficit, when the transpiration rate decreases, and the cell-to-cell pathway, which is mainly regulated by aquaporins, becomes dominant [11]. Aquaporins are key regulators of transcellular water transport and membrane permeability, and their important role in the regulation of plant water status has been demonstrated in many studies [12,13,14,15]. Changes in the functional activity, gene expression level and abundance of aquaporins in plant tissues and organs form the response of the plant hydraulic system to external influences [16,17,18]. However, within the framework of the composite model of water transport, not all factors and signals that cause the redistribution of water flux contributions between the apoplastic and cell-to-cell pathways have been fully explored. In view of the above, it is conceivable that one such factor could be the rate of water flow through the apoplastic pathway.

At present, the relationship and interaction of water flows along apoplastic and cell-to-cell water transport pathways in whole plants is poorly understood. This is mainly due to the methodological and technical difficulties associated with carrying out such studies. Generally, to quantify the intensity of water transport in plants, a parameter such as hydraulic conductivity is used [3]. Hydraulic conductance is an integral parameter, which reflects the total water transfer intensity in the studied specimen. The measurement of hydraulic conductance has historically been carried out on cut plant organs [12]. In this context, the measurement of hydraulic conductance is not suitable for studying the relationship between apoplastic and cell-to-cell water transport pathways in plant roots because plant damage leads to the disruption of the integrity and functioning of the plant hydraulic system. The maintenance of plant integrity and use of non-invasive methods is therefore an important requirement for studying of the interaction of water transport pathways in plants. To study the interactions of water transport pathways in whole plant roots, a logical approach is to rapidly affect one water transport pathway and observe the response of another water transport pathway in parallel. In view of the above and following the hypothesis that the intensity of apoplastic water transport in roots influences cell-to-cell water transport, the dynamics of cell-to-cell water transport and cell water permeability in the roots of intact maize plants, as well as other physiological parameters of the plants, were studied under rapid blocking of water transport via the apoplastic pathway. To this end, an original methodological and technical approach based on a low-field NMR technique coupled with climatic chambers [11] and using the method of rapid apoplast blockage by insoluble precipitates was applied. This approach allowed us to study the intensity of water transport in whole plant roots under controlled environmental conditions and simultaneous manipulations with plants in real time dynamics.

## 2. Materials and Methods

### 2.1. Plant Material

Maize plants (*Zea mays* L.) of the variety Mashuk were used in the experiments. The seeds of the plants were germinated at a constant temperature of 25 °C for 3 days in a cuvette with CaCl_2_ solution (0.0025 M). Then, seedlings with root lengths of 1 to 2 cm were transferred to special glass capillaries floating in a vertical position and placed in cups of 1 L. Plants in cups were grown in CaCl_2_ solution for 4–5 days in original climatic chambers adapted for the NMR technique [11,19], under a 12 h photoperiod (illumination 15 W m^2^), artificial root aeration, temperatures of 24–25 °C during the day and 20–21 °C at night, 50% humidity and a carbon dioxide concentration of 380–420 ppm. Plant roots with approximately equal root length (about 15 cm) were then placed in special 11 mm diameter tubes containing CaCl_2_ solution adapted for the NMR probe and climatic growth chambers [11]. Each tube contained 15 plants. Tubes in quantities of six to nine were placed in climatic chambers where the plants were kept for the next 24 h under artificial root aeration before the experiments at the above climatic parameters. Prior to the NMR experiment, one of the climatic chambers was placed on the NMR equipment. The tube containing the plants was then placed in the NMR probe so that part of the root suction zone (5–6 cm from the root tip) was within the measurement zone of the NMR probe. Manipulations of the plant roots, such as apoplast blockage and exposure to an aquaporin inhibitor, were performed by adding the necessary solutions to the root medium via an aperture in the base of the tube during the NMR measurements.

### 2.2. Rapid and Partial Blockage of Apoplastic Water Transport Pathway in the Roots of Intact Maize Plants

One of the main methodological objectives was to significantly reduce the rate of water transport through the apoplastic pathway in whole plant roots as rapidly as possible, while simultaneously measuring parameters reflecting the intensity of radial water transport along the cell-to-cell pathway. Firstly, the rapid blockage of the apoplast allowed the registration of an early response in the cell-to-cell water transport, avoiding measurements during the possible development of severe water stress and, to a greater extent, excluding the involvement of chemical signals in the regulation of cell-to-cell water transport. The control of environmental parameters such as temperature, humidity and atmospheric carbon dioxide concentration was also important in this case, as changes in these parameters during the experiment could affect the initial state of the plant hydraulic system. To rapidly reduce the rate of water transport through the apoplastic pathway, an original approach was used. This approach consisted of blocking the apoplast of the outer layers of root cells in whole plants with the smallest insoluble particles (precipitates) that are formed almost instantaneously when solutions of K_4_[Fe(CN)_6_] and CuSO_4_ are mixed. For this purpose, plant roots were alternately incubated in K_4_[Fe(CN)_6_] and CuSO_4_ solutions. The apoplast-blocking procedure was carried out according to the following scheme. Through the bottom aperture of the tube with plants, previously installed in the NMR probe, a small hose was used to drain CaCl_2_ solution, which irrigated the root medium. Then, through the same hose, a solution of K_4_[Fe(CN)_6_] at a concentration of 1 mM and a volume of 25 mL was poured into the tube. The solution volume of 25 mL was sufficient to completely submerge the roots. The roots were exposed to this solution for 3 min. It was assumed that the K_4_[Fe(CN)_6_] solution had time to penetrate the apoplast of only the outer cell layers, mainly the rhizoderm and the epidermis. K_4_[Fe(CN)_6_] solution was then drained and CuSO_4_ was immediately added to the root medium at a concentration of 0.5 mM, also in a volume of 25 mL. The concentration of the K_4_[Fe(CN)_6_] and CuSO_4_ solutions was chosen according to the work of Ranathunge and co-authors [9], in which it was shown that the indicated concentrations were minimal and most effective at forming salt precipitates and obstructing the root apoplast. When CuSO_4_ entered the root apoplast, it interacted with the K_4_[Fe(CN)_6_] and formed insoluble salt particles in the apoplast, leading to the apoplast blockage and a reduction in the rate of water transport through the apoplast. After 5 min, the CuSO_4_ solution was also drained and the initial CaCl_2_ solution was added. Thus, insoluble particles were formed directly in the root apoplast and not embedded there by the action of external forces. The originality of this approach lies in the drastic restriction of water movement into the root of a whole plant without changing the quantitative water content of the root environment. To reduce the risk of toxic effects, the incubation time of the roots in K_4_[Fe(CN)_6_] and CuSO_4_ solutions was minimized to 3 and 5 min, respectively. Preliminary experiments showed that incubation of roots in K_4_[Fe(CN)_6_] and CuSO_4_ solutions separately did not cause changes in D_ef_. Another important factor was the sequence in which the solutions were applied. The roots were first incubated in a K_4_[Fe(CN)_6_] solution, followed by a CuSO_4_ solution, not vice versa. As a result, the Cu^2^⁺ ions could not penetrate deeply into the root, causing toxic effects. This was because the interaction between the CuSO_4_ and the K_4_[Fe(CN)_6_] at the root periphery resulted in the formation of salt crystals almost instantly. These crystals blocked the apoplast of the outer cell layers, preventing the CuSO_4_ solution from penetrating deeply into the root. The formation of apoplast-blocking precipitates was observed by light microscopy. To this end, root sections that were 60 µm thick at a distance of 5–6 cm from the root tip of control and treated plants were prepared using the vibratome Leica VT 1000S (Leica Biosystems, Nußloch, Germany). The sections were examined using a light microscope Leica DM1000 (Leica Biosystems, Germany).

### 2.3. Confocal Microscopy Using Fluorescent Nanoparticles

The decrease in the rate of water transport through the apoplastic pathway was qualitatively estimated using a fluorescent marker in the form of silicate nanoparticles [11]. The size of the nanoparticles was approximately 30 nm. The essence of this approach is to compare the depth of penetration of nanoparticles into the roots of control and treated (after apoplast blockage) plants for the same period of incubation of the plants in solutions containing nanoparticles. An aqueous solution of nanoparticles at a concentration of 6 g/L was used for control and treated plants. After two hours of incubation, the roots of control and treated plants were washed in water for three minutes. Cross-sections were then hand-cut at a distance of 6 cm from the root tip and observed under a LSM780 confocal microscope (Carl-Zeiss, Oberkochen, Germany). The excitation and emission wavelengths used were 430 and 605 nm, respectively.

### 2.4. NMR Measurements of Cell-to-Cell Water Transport in Lateral Direction of the Roots of Intact Plants

The intensity of cell-to-cell water transport and cell water permeability in the roots of maize plants was studied using the spin-echo NMR [20,21]. This method is widely used to study water transport in biological samples, including plant tissues [22,23,24,25]. Its advantages include its non-invasiveness and its ability to separate the contributions of different water transport pathways in plant tissues [26,27,28]. The essence of the method is to observe the translational diffusion of water molecules in root tissue when a pulsed magnetic field gradient is applied to the sample. Using a three-pulse stimulated echo sequence, diffusion decays (DDs) of the water magnetization in the sample were registered [21]. DD is the dependence of the relative stimulated echo signal amplitude R from water molecules on the parameters of a pulse sequence: g—amplitude of pulses of magnetic field gradient, t_d_—diffusion time as the interval between gradient pulses, δ—gradient pulse duration [29]. This dependence is expressed as follows:

(1) $$R=exp(-\gamma^{2}\delta^{2}g^{2}{(t}_{d}-\delta /3)D_{ef})$$

where R is the relative echo amplitude, which equals the ratio of echo amplitudes in the presence and absence of the magnetic field gradient, R = A(g)/A(0); γ—proton gyromagnetic ratio (the constant is equal to 2.67 × 10^8^ T^−1^s^−1^); D_ef_—effective water self-diffusion coefficient. D_ef_ was calculated by fitting the initial part of DDs with Equation (1). The initial part of DD (at g → 0) contains information on the magnitude of the diffusional displacement of molecules, regardless of the type of diffusion propagator. In this case, the diffusion attenuation of the initial part of DDs within one order of magnitude is satisfactorily approximated by the exponential (1). In this case, D_ef_ can be considered as the average self-diffusion coefficient. Measurements of average effective self-diffusion coefficients are convenient because they do not require measurements in a wide range of diffusion attenuation, and this significantly reduces the duration of the experiment. It also does not require pulses with high-intensity magnetic field gradients. Thus, D_ef_ was the main parameter used in this study to characterize the intensity of cell-to-cell water transport. The D_ef_ value, measured at the maximum possible diffusion observation time (long t_d_), directly correlates with the water permeability of cells and, consequently, with the intensity of cell-to-cell water transport [11,24]. Usually, the diffusion time is selected in a preliminary manner and mainly depends on the structure of the investigated sample, in particular on the cell size, and is limited by the required amplitude of the NMR signal. In our case, in the suction zone of maize roots, the long t_d_ was 700 ms. Thus, to evaluate rapid changes in the intensity of cell-to-cell water transport, it was sufficient to measure D_ef_ at one diffusion time point (long td), since in this case the measurement process took less time. Calculations of absolute values of cell water permeability P in roots and average cell size were based on the full dependence of D_ef_ on t_d_ in roots in the range of t_d_ from 20 ms to 700 ms [28,29]. NMR experiments were carried out at 25 °C on the time domain ^1^H NMR equipped with a digital analyzer “Spin Track” (Resonance Systems Ltd., Yoshkar-Ola, Russia) operating at 19.1 MHz. The pulsed magnetic field gradient was applied in the lateral direction of root suction zone and water diffusion was observed in this direction, accordingly. This spin-echo NMR approach allowed the study of the intensity of water transport in whole plant roots under controlled environmental conditions and simultaneous manipulations with plants in real-time dynamics. The main limitations, but not disadvantages, of the spin-echo NMR technique used are the impossibility of studying water transport parameters using a single plant (root) due to low sensitivity, as well as the impossibility of measuring the self-diffusion coefficient separately for different tissues. The resulting self-diffusion coefficient is averaged over all root tissues.

### 2.5. Estimation of Aquaporins’ Contribution to Cell-to-Cell Water Transport by Inhibitory Analysis

The contribution of aquaporins to the regulation of cell-to-cell water transport in response to the apoplast blockage was estimated using a mercuric chloride inhibitor assay. Mercuric chloride is a widely used inhibitor of aquaporins which can significantly reduce the water permeability of cell membranes [19,30,31]. For aquaporin inhibition, a mercuric chloride solution at a concentration of 200 μM/L was poured into the root medium instead of the CaCl_2_ solution through the aperture in the base of the tube with plants while they were in the NMR probe. Twenty minutes after addition, the mercuric chloride solution was replaced with the initial CaCl_2_ solution. While the plants were exposed to mercuric chloride, NMR measurements of water transport parameters were performed. This allowed real-time monitoring of the efficiency of the mercuric chloride effect on cell-to-cell water transport intensity. In order to eliminate the inhibitory effect of mercuric chloride on water transport, the roots were incubated for 20 min in a solution of 5 mM β-mercaptoethanol.

### 2.6. RNA Extractions, cDNA Synthesis, and PCR Reactions

RNA extractions were performed using the plant primary root absorption zone where the NMR parameters of water transport were measured. The preliminary cutting of the sample and freezing in liquid nitrogen took place at about 12 am; 2 cm long root segments from the absorption zone (5–6 cm from the root tips) were used. The samples were fixed in liquid nitrogen at the moment when, according to NMR measurements, the intensity of cell-to-cell water transfer was lowest after the apoplast blockage. Roots from control plants were fixed at the same time. For each biological replication, root and leaf segments of total mass approximately 100 mg were used from three different plants. RNA extractions were performed using the RNeasy Plant Mini Kit, following the manufacturer’s instructions (Magen, Guangzhou, China). RNA yield and purity was estimated using a Nanodrop TM1000 spectrophotometer (Thermo Fisher Scientific Inc., Waltham, MA, USA). RNA integrity was verified on agarose gels. cDNA synthesis and PCR reactions were performed according to the protocols described in [11]. Control and treated samples were analyzed in three technical and three biological replicates. Plasma membrane-localized aquaporins of maize plants likely to transport water were selected according to the literature data and our previously obtained data. Primers for 8 target and 2 reference genes (Supplementary Table S1) were designed using CLC Genomics Workbench 8 Software and synthesized by Evrogen (Moscow, Russia). For normalization in the qPCR analysis, the normalization factor (NF) was used. NF was calculated based on the geometric mean of two selected reference genes.

### 2.7. Estimation of Relative Transpiration Dynamics in Response to Apoplastic Water Transport Blockage in the Roots

The short-time dynamics of the relative transpiration rate in plants were estimated by the weighting method. To achieve this, portable scales (Ohaus PA214C, Ohaus Corporation, Parsippany, NJ, USA) were placed under the tube with plants located in the climate chamber. The design of the climate chambers made it possible not to remove the tubes of plants from the chambers when placing them on the scales and not to change the climatic parameters of the environment. Each tube contained three plants. In the preliminary stage, 2 h before the measurements, the open surface of the tube containing the plants was covered with parafilm to prevent the evaporation of water from the non-leaf surface. The weight of the tube with plants was then measured in 5 min intervals. During the first 30 min, the weight loss of the tube was measured under control conditions. Then, without moving the plants and scales, the root apoplast blocking reaction was carried out by adding the K_4_[Fe(CN)_6_] and CuSO_4_ solutions to the root medium, and for the next 2 h, the scales continued to be recorded, also at 5 min intervals. During the measurements, the tube with the plants did not touch the edges of the climate chamber. Thus, the dynamics of the change in the mass of the sample tube resulting from the evaporation of water from the leaf surface revealed the response of transpiration to the blockage of apoplastic water transport in the roots.

### 2.8. Measurements of Root Growth Under Apoplast Blockage

To determine the effect of apoplast blockage on root growth, root elongation was measured dynamically before and after the apoplast blockage. To determine root elongation values, the roots were photographed with a camera through the transparent walls of the tubes with plants before and after the apoplast-blocking procedure. The images obtained were analyzed using ImageJ 1.43m. Six plants were used in each of three biological replicates.

### 2.9. Statistics

All experiments were performed with 3 to 5 biological replicates. Statistical analysis of the data was performed using one-way analysis of variance (one-way ANOVA followed by Tukey’s test at α = 0.05). Comparison of mean values between control and treatment groups was performed using the *t*-test at *p* = 0.05. Statistical analysis was conducted using OriginPro 8.5 (OriginLab Corp., Northampton, MA, USA). The calculation results are shown in the figures as the mean values and standard deviations. Differences between means were considered significant at α < 0.05.

## 3. Results

### 3.1. Checking of Blockage of the Apoplastic Water Transport Pathway

**Figure 1:**
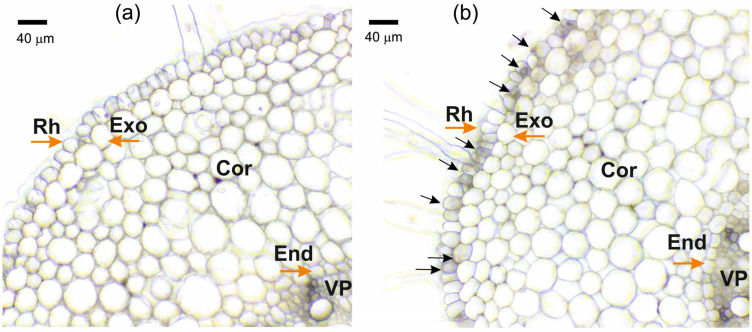
Cross-sections of maize roots from the suction zone: (**a**) Control (without apoplast blockage); (**b**) after apoplast blockage caused by application of K_4_[Fe(CN)_6_] 1 mM for 3 min and CuSO_4_ 0.5 mM for 5 min to roots of whole plants. Rh—rhizodermis; Exo—exodermis; Cor—cortex; End—endodermis; VP—vascular parenchyma. Black arrows indicate the formation of insoluble precipitate suspensions and aggregates. Brown arrows indicate different root tissues.

When solutions of K_4_[Fe(CN)_6_] and CuSO_4_ were mixed, the almost instantaneous formation of salt crystals in the free solutions was easily observed by optical microscopy. However, it was more difficult to observe the formation of individual salt crystals in the apoplast of the outer layers of root cells after the blocking procedure. Instead of individual particles, the formation of particle suspensions and aggregates was observed, which covered the root periphery and were observed in the apoplast of the outer layers of the root cells (Figure 1b). This particle suspension became more apparent and distinct when roots were incubated for longer periods in K_4_[Fe(CN)_6_] and CuSO_4_ solutions.

**Figure 2:**
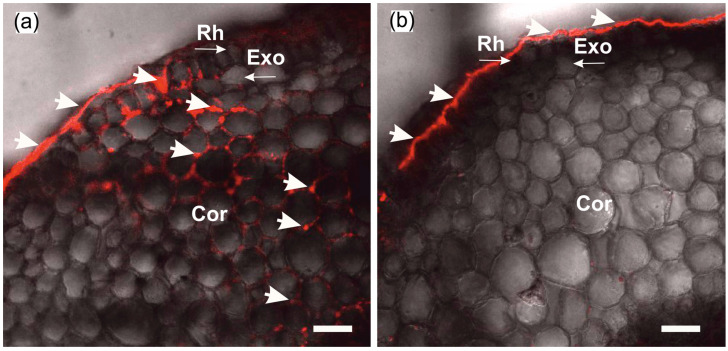
Cross-sections of maize roots from suction zone after 2 h of incubation of roots of intact plants in solutions with fluorescent nanoparticles at concentration of 6 g/L: (**a**) Incubation of roots under normal conditions (without apoplast blockage); (**b**) incubation of roots after apoplast-blocking procedure. Rh—rhizodermis; Exo—exodermis; Cor—cortex; thick arrows indicate fluorescent nanoparticles in the root apoplast. Thin arrows indicate different root tissues. Bars = 40 µm.

The formation of insoluble particles or their suspension in the apoplast at the root periphery does not yet prove that water transport via the apoplastic pathway is blocked. For this reason, the extent of blockage of water transport via the apoplast pathway was assessed prior to measurements of cell-to-cell water transport dynamics. To this end, a comparative assessment of the depth of penetration of fluorescent nanoparticles into the root apoplast was carried out after incubation of control and treated plants (after blocking the apoplast) for two hours in aqueous solutions containing nanoparticles. It was assumed that the higher the rate of water flow through the apoplast, the greater the depth of penetration of nanoparticles into the root apoplast. In preliminary experiments, it was demonstrated that nanoparticles were distributed only in the root apoplast without penetrating the cells, at least during the 2 h incubation of roots in the nanoparticle solution. As a result, in control plants (without apoplast blockage), nanoparticles were registered in the root apoplast, starting from the periphery towards the inner layers of the cortex (Figure 2a). In contrast, in the treated plants, the nanoparticles did not penetrate the apoplast, even that of the outer cells of the roots (Figure 2b). This indicates that after the apoplast-blocking procedure, the water flux through the apoplast was significantly reduced.

### 3.2. Dynamics of Cell-to-Cell Water Transport in the Roots in Response to Apoplast Blockage

**Figure 3:**
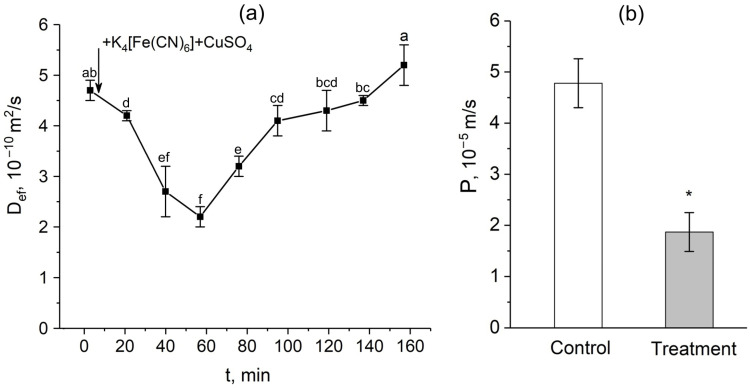
Dynamics of the effective self-diffusion coefficient (D_ef_) and cell water permeability (P) in the roots of whole maize plants after the root apoplast blockage. (**a**) Short-term dynamics of D_ef_ after the apoplast blockage. The black arrow indicates the time point of the apoplast blockage by application of K_4_[Fe(CN)_6_] 1 mM for 3 min and CuSO_4_ 0.5 mM for 5 min. D_ef_ was measured at a diffusion time (t_d_) of 700 ms; (**b**) cell water permeability P in control (without apoplast blockage) (white column) and after 40 min of the apoplast-blocking procedure (grey column). Bars show ±SD (*n* = 3). For figure (**a**), mean separation was determined by one-way ANOVA followed by Tukey’s test at α = 0.05. Different letters indicate significant differences between D_ef_ values at different times after the apoplast blockage. * indicates significant difference between control and treatment according to *t*-test at α = 0.05.

After the apoplast blockage, there were rapid and significant changes in the intensity of water transport through the cell-to-cell pathway. Figure 3a shows the dynamics of changes in the effective self-diffusion coefficient (D_ef_) of water in plant roots after apoplast blockage. As mentioned above, D_ef_ reflects the intensity of cell-to-cell water transport and correlates with the water permeability of the cells. According to the data obtained, there was a decrease in D_ef_ 15 min after the apoplast blockage compared to the control value (before the apoplast blockage). The decrease in D_ef_ continued for approximately 40 min. During this time, the level of D_ef_ decreased by approximately 2.5-fold. A detailed study of the dependence of D_ef_ on the diffusion time t_d_ showed that 40–50 min after the apoplast blockage, when D_ef_ reached its minimum value, the cell water permeability P decreased compared to the control value before the apoplast blockage (Figure 3b). Interestingly, after the reduction stage, there was a sharp and rapid increase (recovery) in D_ef_, which ultimately surpassed the initial values (Figure 3a).

### 3.3. Root Aquaporin Involvement in Response to Apoplast Blockage

**Figure 4:**
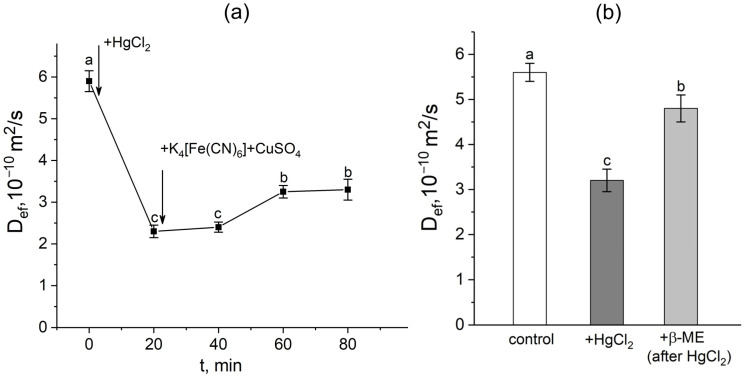
Effect of the apoplast blockage, HgCl_2_, and β-mercaptoethanol (β-ME) treatments on the effective self-diffusion coefficient (D_ef_) in the roots of whole maize plants. (**a**) Short-term dynamics of the effective self-diffusion coefficient (D_ef_) in response to the root apoplast blockage after inhibition of water transport through aquaporins by HgCl_2_. Black arrows indicate the time point of aquaporin inhibition resulting from application of HgCl_2_ to the roots and the apoplast blockage resulting from application of K_4_[Fe(CN)_6_] 1 mM for 3 min and CuSO_4_ 0.5 mM for 5 min; (**b**) effective self-diffusion coefficient (D_ef_) in control (without treatments) (white column), after application of HgCl_2_ (0.2 mM, 20 min) (dark grey column), and after application of β-mercaptoethanol (5 mM, 20 min) following HgCl_2_ treatment (light grey column) D_ef_ was measured at a diffusion time (t_d_) of 700 ms. Bars show ±SD (*n* = 3). Mean separation was determined by one-way ANOVA followed by Tukey’s test at α = 0.05. Different letters indicate significant differences between D_ef_ values at different times and treatments.

In the first step, a mercuric chloride inhibition assay was used to investigate the contribution of aquaporins in response to the apoplast blockage. Plant roots were exposed to mercuric chloride in three different situations: (1) before the apoplast blockage; (2) after the apoplast blockage when D_ef_ was significantly reduced; and (3) after the apoplast blockage at the D_ef_ recovery stage. It is worth noting that the time of the beginning of roots’ exposure to the mercuric chloride was chosen on the basis of the dynamics of the D_ef_ obtained in real time. In the first variant, 20 min of exposure to mercuric chloride resulted in the expected and significant decrease (by 40–60%) of D_ef_ (Figure 4a,b). However, after exposure to mercuric chloride, the apoplast blockage did not result in a decline in D_ef_. Instead, an increase in D_ef_ was observed. The inhibitory effect of HgCl_2_ on transcellular water transport in roots was largely reversed by 20 min exposure of HgCl_2_-treated roots to 5 mM β-mercaptoethanol solution (Figure 4b).

**Figure 5:**
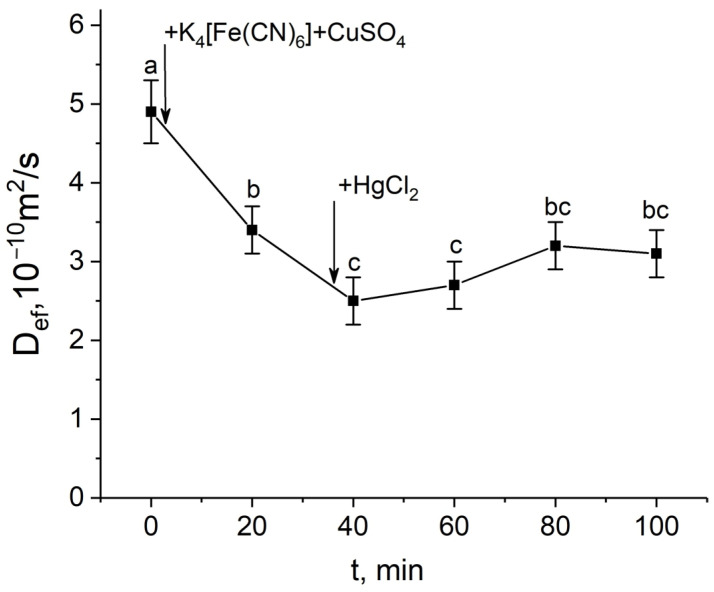
Short-term dynamics of the effective self-diffusion coefficient (D_ef_) in the roots of whole maize plants in response to inhibition of aquaporins by HgCl_2_ after root apoplast blockage. D_ef_ was measured at a diffusion time (t_d_) of 700 ms. Black arrows indicate the time point of apoplast blockage by application of K_4_[Fe(CN)_6_] 1 mM for 3 min and CuSO_4_ 0.5 mM for 5 min to the roots, and aquaporin inhibition by application of HgCl_2_. Bars show ±SD (*n* = 4). Mean separation was determined by one-way ANOVA followed by Tukey’s test at α = 0.05. Different letters indicate significant differences between D_ef_ values at different times.

In the second variant, exposure to mercuric chloride after blocking the apoplast at the time of a significant decrease in D_ef_ had a small additive effect, but further D_ef_ values did not change and the D_ef_ recovery phase, always observed in control plants, was absent (Figure 5).

**Figure 6:**
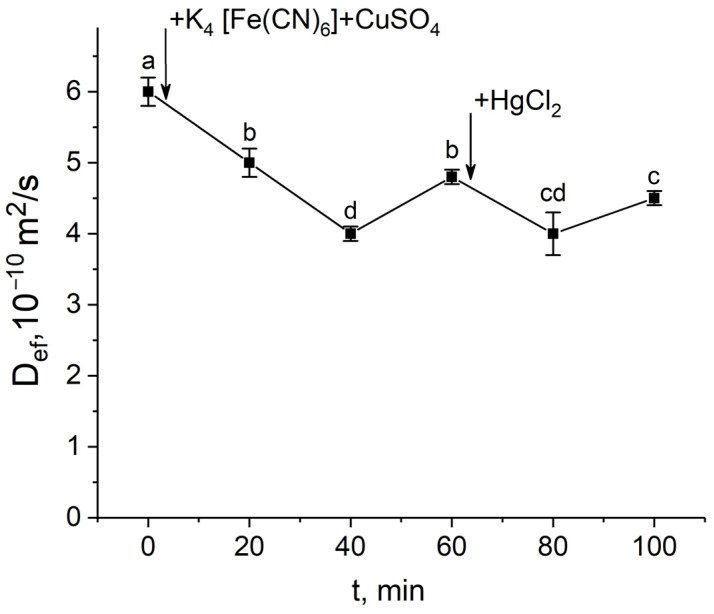
Short-term dynamics of the effective self-diffusion coefficient (D_ef_) in roots of whole maize plants in response to inhibition of aquaporins by HgCl_2_ at the D_ef_ recovery stage after root apoplast blockage. D_ef_ was measured at a diffusion time (t_d_) of 700 ms. Black arrows indicate the time point of apoplast blockage resulting from applying K_4_[Fe(CN)_6_] 1 mM for 3 min and CuSO_4_ 1 mM to roots for 5 min, and aquaporin inhibition resulting from application of HgCl_2_. Bars show ±SD (*n* = 3). Mean separation was determined by one-way ANOVA followed by Tukey’s test at α = 0.05. Different letters indicate significant differences between D_ef_ values at different times.

In the third variant, exposure to the mercuric chloride at the D_ef_ recovery stage after the apoplast blockage resulted in a decrease in D_ef_, i.e., in this case, the inhibitory effect of mercuric chloride was revealed (Figure 6).

**Figure 7:**
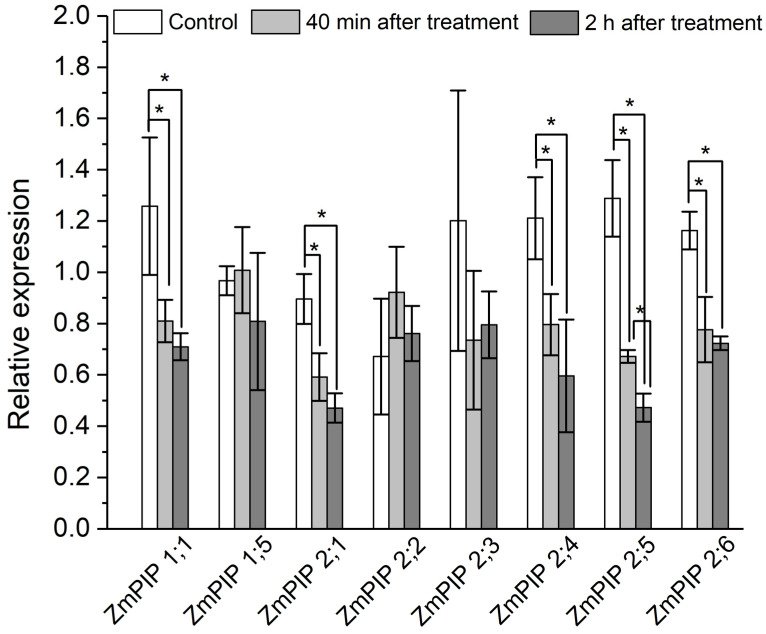
Relative expression levels of plasma membrane aquaporin (AQP) genes in roots of maize plants in control (without the apoplast blocking) (white columns), 40 min after the apoplast blockage (light grey columns) and 2 h after the apoplast blocking (dark grey columns). Bars show ±SD (*n* = 6). For each AQP gene, mean separation between control and treatment values was determined by a *t*-test at α = 0.05. * indicates significant difference between relative expression values.

To elucidate the role of aquaporins, in addition to experiments with the aquaporin inhibitor mercuric chloride, the expression level of plasma membrane aquaporin genes in the suction zone of plant roots was investigated in response to the apoplast pathway blocking. The results showed that 40 min after the apoplast blockage, when a significant decrease in D_ef_ and water permeability of P cells, as measured by NMR, was achieved, the relative expression level of most of the aquaporin genes decreased. In particular, a decrease in the expression level of the PIP1 and PIP2 isoforms ZmPIP1;1, ZmPIP2;1, ZmPIP2;4, ZmPIP2;5, ZmPIP2;6 was observed (Figure 7). The expression levels of the other aquaporins (ZmPIP1;5, ZmPIP2;2, ZmPIP2;3) did not alter in response to the apoplast blockage. Two hours after the apoplast blockage, the relative levels of aquaporin gene expression remained below control values (Figure 7). Thus, the regulation of aquaporins in response to the blockage of the apoplastic water transport pathway in roots occurred at the transcriptional level.

### 3.4. Dynamics of Transpiration and Root Growth After Apoplast Blockage

**Figure 8:**
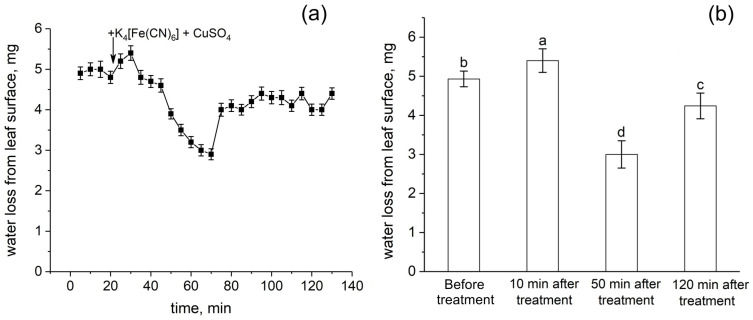
Dynamics of relative transpiration (water evaporation from the leaf surface) after the root apoplast blockage (treatment). (**a**) Detailed dynamics of relative transpiration after the apoplast blockage. The black arrow indicates the time point of the apoplast blockage resulting from applying K_4_[Fe(CN)_6_] 1 mM for 3 min and CuSO_4_ 0.5 mM to the roots for 5 min. (**b**) Relative transpiration in control (before the apoplast blockage) and at 10 min, 50 min, and 120 min after the apoplast blockage. Bars show ±SD (*n* = 3). Different letters indicate significant differences between relative transpiration values at different times after apoplast blockage.

The action of transpiration is considered to create a hydrostatic pressure gradient along the plant and is one of the driving forces of water transport. Transpiration causes passive water transport along the root apoplast. In this respect, the response of transpiration to the root apoplast blockage is an important indicator of the rate of apoplastic water transport in the root. Figure 8 shows the detailed dynamics of the relative level of transpiration after the root apoplast blockage. Interestingly, the transpiration rate increased during the first 10 min after the apoplast blockage (Figure 8a,b). This was followed by a decrease in transpiration of about 45% within 40 min. However, the decrease in transpiration then reverted to an increase (recovery) that occurred much more rapidly. In total, the recovery of transpiration level after the drop was about 20%. The transpiration rate then remained continuously lower than the initial values (before the apoplast blockage) by 15%. Thus, the transpiration rate clearly responded to the apoplast blockage in plant roots and the dynamics of transpiration was complex.

**Figure 9:**
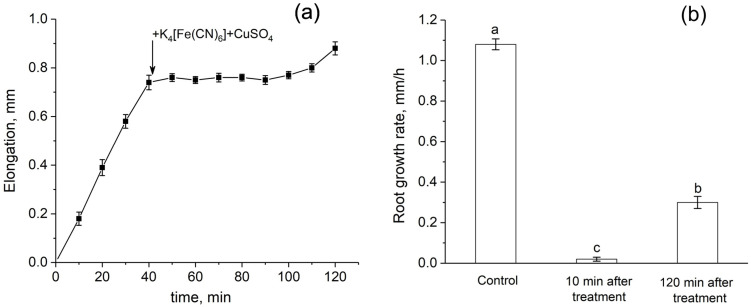
Dynamics of root elongation and root growth rate before and after the root apoplast blockage. (**a**) Detailed dynamics of root elongation before and after the apoplast blockage. The black arrow indicates the time point of the apoplast blockage that occurred as a result of applying K_4_[Fe(CN)_6_] 1 mM for 3 min and CuSO_4_ 0.5 mM to the roots for 5 min. (**b**) Root growth rate in control (before the apoplast blockage), at 10 min, and at 120 min after the apoplast blockage. Bars show ±SD (*n* = 3). Different letters indicate significant differences between growth rate values.

Root growth was almost completely inhibited immediately after the apoplast blockage (Figure 9a,b). Recovery of growth rate started only 2 h after the blockage. One day after the treatment, the root growth rate of treated plants did not differ from that of control plants.

## 4. Discussion

The study of the relationship and redistribution of contributions of water transport pathways in plant roots, particularly the apoplastic and cell-to-cell pathways, is important for understanding the functioning of the root hydraulic system under different conditions. The main idea of this study was formed in an attempt to experimentally observe the behaviour of cell-to-cell water transport in whole plant roots when the root apoplast is blocked. One of the main hypotheses was that the intensity of the apoplastic water flux in the root itself could be a factor in the regulation of cell-to-cell water transport and cell water permeability. The peculiarity of this study from a methodological point of view was the possibility of rapidly reducing water transport via the apoplastic pathway by insoluble particles, while simultaneously observing the intensity of cell-to-cell water transport and the water permeability of root cells. The reaction between K_4_[Fe(CN)_6_] and CuSO_4_ with formation of insoluble Cu_2_[Fe(CN)_6_] for the apoplast blockage in plant roots was also used in other studies. In the study conducted by Ranathunga et al., it was demonstrated that the blockage of the apoplastic pores in rice root segments with precipitates caused a three- to four-fold reduction in hydraulic conductivity of the outer part of rice roots [9]. These results indicated that despite the presence of an exodermis with Casparian bands, most of the water in rice roots moves via the apoplastic pathway rather than the cell-to-cell route. In another similar work, blocking the apoplast with insoluble particles in chickpea roots resulted in a significant reduction in the transpiration rate. Based on this result, the authors concluded that the apoplastic pathway of water transport is dominant [32]. In the above-mentioned works, the apoplast blockage was performed over the total root volume and lasted several hours, and the hydraulic conductivity of root segments was measured by the classical method using a pressure chamber. An important difference in the present study was that the experiments were performed on whole plants and the method used involved rapid and partial blocking of the apoplast in roots. In addition, the non-invasive measurement of water transport parameters in the roots was carried out dynamically during the manipulation of plant roots. The approach used allowed us to obtain the dynamics of the intensity of cell-to-cell water transport in the lateral direction of the roots and the transpiration rate in response to blockage of the apoplastic pathway of water transport in the roots. In addition, data on the contribution of aquaporins in roots in response to the apoplast blockage were obtained.

As a result of the formation of insoluble particles in the root periphery and in the apoplast of the outer root cells during the blocking procedure, the water supply to the root is significantly reduced, mainly through the apoplastic pathway. Firstly, due to the short incubation time of roots in K_4_[Fe(CN)_6_] and CuSO_4_ solutions, insoluble salt particles have time to form, mainly in cell walls and intercellular spaces in peripheral root cells (Figure 1), creating a barrier that prevents water flow in the apoplast. Given that the apoplast pathway is continuous from the root periphery to the endodermis, obstruction of the apoplast at the root periphery should result in the blockage of water flow along the entire length of the apoplast pathway. Secondly, the rate of water flow along the apoplastic pathway under normal conditions is known to be several times higher than the rate of water flow along the cell-to-cell pathway, but at the same time the apoplastic pathway, with a relatively small volume fraction, provides a contribution of up to 50% or more to the total hydraulic conductivity of the root [10]. Therefore, from a physical and mechanical point of view, the rate of water flow through the apoplastic pathway is mainly affected during the rapid blocking procedure. Experimental evidence for a reduction in the rate of water flow through the apoplast is provided by the use of fluorescent nanoparticles, which did not penetrate the apoplast, even in the outer root cells, within two hours after the apoplast-blocking procedure (Figure 2b). These results also suggest that the decrease in water transport through the apoplast occurs immediately after blocking and continues for at least two hours. Otherwise, if the rate of water flow through the apoplast after blocking remained at or near the initial level for some time, the fluorescent particles would have had time to penetrate the root apoplast. However, despite the fact that the fluorescent nanoparticles did not penetrate the root apoplast after blocking, it cannot be concluded that water supply to the root via the apoplastic pathway was totally blocked. Root growth, as a parameter that is strongly dependent on water supply, was also significantly inhibited in response to the apoplast blockage (Figure 9a,b). In addition, the decrease in transpiration rate after the apoplast blockage (Figure 8a,b) may also be indicative of a decrease in the rate of water transport along the apoplastic pathway in the roots. Regarding the effect of the apoplast-blocking procedure itself on the cell-to-cell water transport pathway, it should be noted that a decrease in the rate of cell-to-cell water transport in peripheral root cells, particularly in rhizodermal and epidermal cells, cannot be excluded. However, due to the small area in the root cross-section, these cells make a minor contribution to the diffusion NMR experiment, in which the major NMR signal is contributed by water from the root inner cells, in particular the cells of the root cortex, which are free of blocking particles.

The results of diffusion NMR experiments showed that the intensity of the cell-to-cell water transport and the cell water permeability P in the root suction zone responded clearly to the blocking of the apoplastic water transport in the root (Figure 3a,b). The dynamics of the change in D_ef_ were two-stage and multidirectional (Figure 3a). At the first stage, there was a decrease in D_ef_, which then changed into an increase (recovery) in D_ef_, which rose to the initial values and then surpassed them. The results of experiments with the aquaporin inhibitor mercuric chloride and analysis of aquaporin gene expression showed that aquaporins are involved in the observed decrease in cell water permeability in response to the apoplast blockage. Firstly, this is evidenced by the fact that after a preliminary reduction in cell water permeability by mercuric chloride, subsequent apoplast blockage had no effect on the intensity of the cell-to-cell water transport (Figure 4). Secondly, at the time point corresponding to the greatest decrease in D_ef_ and P (approximately 40 min after the apoplast blockage) (Figure 3a,b), the expression of most of the aquaporin genes analyzed was lower than the control level (Figure 7). It is worth noting that the focus of the analysis of aquaporin gene expression was on plasmalemma aquaporins, because the plasmalemma is the barrier that determines the cells’ final permeability to water during cell-to-cell water transport. The greatest decrease in expression was observed for ZmPIP2;5, which is one of the PIP2 isoforms of aquaporins most actively involved in the regulation of water transport in both roots and leaves of maize plants [33,34,35]. Thus, blocking the apoplast and reducing the rate of water transport through the apoplastic pathway resulted in a relatively rapid decrease in the intensity of aquaporin-mediated cell-to-cell water transport. Therefore, a logical question arises—what is the factor or signal for the reduction in cellular water permeability and/or aquaporin activation at the initial stage after blockage of water transport through the root apoplast? One such factor could be the physical effect of the apoplastic water flow on the rate of cell-to-cell water transport, since the apoplastic and cell-to-cell water transport pathways are overlapping. Based on the obtained results, it can be assumed that the higher rate of water flow along the apoplast, the higher the entrainment of water moving along the cell-to-cell pathway, and vice versa. This is also supported by the “hydraulic fuse” theory [36]. According to this theory, the permeability of the plasmalemma and the intensity of cell-to-cell water transport can be regulated by the distance between the plasmalemma and the cell wall and by the difference in water potentials between the cell wall and the protoplast. This suggests that the plasmalemma can “sense” the water status in the cell wall.

It is important to note that no alterations in the cell-to-cell water transport were detected during the first ten minutes following the apoplast blockage. However, the formation of blocking particles and the restriction of water entry occurred almost immediately after the blocking procedure. The pattern of DDs in the roots remained unchanged during this period. A significant decrease in D_ef_ with respect to the control (before the apoplast blockage) occurred not earlier than 10–15 min after blocking (Figure 3a). Interestingly, the transpiration rate did not decrease during the first ten minutes after the apoplast blockage but, on the contrary, increased (Figure 8a,b). These results indicate that some time is needed to activate the cell-to-cell water transport and to reduce cell membrane permeability after the apoplast blockage, which seems to be necessary to change the functional state of water-conducting elements, particularly aquaporins. This delayed response may also be related to a gradual rather than a rapid decrease in the rate of water transport along the apoplastic pathway within the root cortex. Although the apoplast blockage on the other part of the root is rapid, inside the root, the reduction in the rate of water transport along the apoplast may be gradual, reaching a threshold or trigger value. It should be noted that it is methodologically difficult to determine the rate of reduction in water flux along the apoplast inside the root while limiting water input from the outside. If, at the same time, there is no compensation for the loss of water flow through the apoplast to meet transpiration demand, for example, by increasing the rate of the cell-to-cell water transport, then we should expect changes in the hydrostatic pressure gradient in the extracellular water continuum along the whole plant. A change in the hydrostatic pressure gradient may provide a hydraulic signal that can activate water channels in a short time period [37,38,39]. For example, in a study by Vandeleur and co-authors, a decrease in cell water permeability in roots 5 min after leaf detachment was attributed to the closure of root aquaporins by a gating mechanism [40] triggered by a hydraulic signal [41]. Thus, the rate of apoplastic water flux can modulate the intensity of the cell-to-cell water transport, both through functional activity of aquaporins and through physical effects.

The effect of a short-term increase in transpiration rate after the apoplast blockage (Figure 8a) can also be caused by a hydraulic signal and may be the reason for a prolonged decrease in water flow in the root apoplast. It is important to note that a similar short-term increase in transpiration rate to stress factors was reported previously in other studies. In the work of Hachez and co-authors, the effect of PEG on roots resulted in a short-term increase in transpiration [42]. In our previous study, the application of 10% PEG solution to the roots also resulted in a sharp increase in transpiration during the first 5 min [11]. From a physiological point of view, this transpiration behaviour can be explained by the plant trying to conserve the amount of water absorbed by the roots during the early phase of water stress.

The obtained D_ef_ dynamics were characterized by a rapid, almost jump-like switch to the stage of D_ef_ increase (recovery), which occurred 40–50 min after the apoplast blockage. The recovery stage of the intensity of the cell-to-cell water transport could be explained by the fact that the particles blocking the apoplast are eventually washed out of the outer layers of the root cells, leading to the restoration of the water flow rate along the apoplast. However, the particle washout effect cannot occur rapidly, and therefore a sudden transition from a decrease to an increase in D_ef_ dynamics is probably not possible. The experiment with fluorescent nanoparticles also demonstrated that the blocking of the apoplastic water transport is maintained for 2 h. Consequently, the recovery phase of D_ef_ and P is rather regulatory in nature. It is also of interest to note that the transpiration rate during the D_ef_ recovery stage partially but rapidly recovered (Figure 8a,b). It can be assumed that the recovery stage in the dynamics of cell-to-cell water transport and transpiration rate indicates the development of a physiological compensatory effect, as a result of which, when the intensity of apoplastic water transport decreases, the cell-to-cell pathway of water transport becomes dominant, partially compensating for transpiration demands. Similar behaviour in the dynamics of D_ef_ and P of root cells was shown in a previous study of the dynamics of water transport in maize roots under water stress induced by exposure of roots to 10% PEG 6000 solution [11]. However, when roots were exposed to osmotic stress, the water transport response in roots was less pronounced and the amplitude of changes in D_ef_ and P was smaller than when the apoplast was blocked. This may be due to the fact that additional and uncontrolled factors arise during PEG exposure. In particular, compared to the method of rapid blocking of the apoplast, the use of osmotic stress causes an additional osmotic force directed against the hyperosmotic solution from the root, which can affect both the water in the apoplast and the water inside the cells. At the same time, in the case of osmotic application, it is difficult to estimate the direction and rate of change of the water flow in the apoplast in short-term dynamics. The use of osmotic solutions to simulate water deficit causes side effects, such as changes in cell and tissue morphology due to dehydration [43]. It is worth noting that in the present work, the microscopy data did not show changes in the size and shape of root cells (Figure 1a,b), which are usually observed when roots are exposed to hyperosmotic solution. The results of NMR experiments with aquaporin inhibition at the time of greatest D_ef_ decrease (Figure 5) and at the time of D_ef_ recovery (Figure 6) can support the physiological regulation of the intensity of cell-to-cell water transport at the recovery phase. The results showed that in these variants, the D_ef_ recovery stage did not occur or was suppressed. However, two hours after the apoplast blockage, i.e., at the stage of recovery of D_ef_ and transpiration rate, the expression level of most aquaporin genes remained below control values (before the apoplast blockage) (Figure 7). This may indicate that additional processes can be involved in the regulation of aquaporins and, more commonly, in the regulation of cell-to-cell water transport during the recovery stage. In particular, aquaporin regulation may occur through phosphorylation/dephosphorylation [44], pH-dependent gating of aquaporins through rearrangement of conserved residues [45], and the heteromerization of PIP aquaporins [46]. The symplastic pathway through the plasmodesmata, which includes cytoplasmic diffusion as well as mass flow along the vacuolar cell-to-cell pathway, also contributes to the total cell-to-cell water transport [47,48]. Overall, the recovery stage of cell-to-cell water transport intensity following apoplast blockage, which may be based on a compensatory mechanism, needs to be investigated in more detail. The factors and signals involved in the rapid change in cell-to-cell water transport intensity during the recovery stage are not yet clear.

## 5. Conclusions

In conclusion, the dynamics of cell-to-cell water transport during the blocking of the apoplastic water transport pathway in roots of whole maize plants was investigated using a non-invasive spin-echo NMR method and an original method of rapid and partial apoplast blockage. The temporal parameters of the early response of cell-to-cell water transport and transpiration were characterized, and the role of aquaporins in the response to blocking the apoplastic water transport pathway was demonstrated. The results showed that the rate of apoplastic water flux and the state of water in the apoplast can be factors in the regulation of water transport along the cell-to-cell pathway. The novelty and importance of this study lies in the fact that it allows us to expand our understanding of the mechanisms of regulation of the root hydraulic system in the whole plant in terms of the interdependence, or interaction, of different water transport pathways. However, some of the existing and emerging questions remain unsolved. For example, it is still not clear what contributes most to the decrease in the rate of cell-to-cell water transport in the early stage after the apoplast blockage—the direct physical effect of water flow through the apoplast on the cell-to-cell pathway, or the indirect effect via aquaporins regulation? And how can these two factors be separated? The contribution of the symplastic pathway, which is part of the cell-to-cell pathway, in the water transport response is also unclear. It would also be interesting to compare the dynamics of water transport along the cell-to-cell pathway when the apoplastic pathway is blocked under initially normal conditions and under conditions of water deficiency.

## Data Availability

Data supporting this study are included within the article.

## Supplementary Materials

The following supporting information can be downloaded at https://www.mdpi.com/article/10.3390/cells14120902/s1, Table S1: Primers for target PIP aquaporin genes.

## Abbreviations

The following abbreviations are used in this manuscript:

D_ef_	Effective diffusion coefficient of water.
DD	Diffusional decay.
P	Diffusional water permeability of cells.
