# Supplementary material for: The Dynamics of Cell-to-Cell Water Transport and the Involvement of Aquaporins in Response to Apoplast Blockage in the Roots of Intact Maize Plants
Source: Cells. 2025 Jun 14;14(12):902. doi: 10.3390/cells14120902 (PMC12190459; doi:10.3390/cells14120902)
Supplement: Supplementary file 1 [file cells-14-00902-s001.zip › cells-3645615-supplementary.pdf]

Table S1. Primers for target PIP aquaporin genes and two reference genes (ZmFPGS, ZmMEP).

Gene	Primer name	Sequence 5' -> 3'	accession number
ZmPIP1;1	PIP1;1_F	gccgtaatttacaaccagcac	00001d002690
	PIP1;1_R	acctactcttgaacgggatcg	
ZmPIP1;5	PIP1;5_F	gccagttcagctagccatca	00001d051872
	PIP1;5_R	caccgtaccaaaacccaagc	
ZmPIP2;1	PIP2;1_F	agttcgctgccaaggactac	00001d019563
	PIP2;1_R	gccaccgtgatgtacaggaa	
ZmPIP2;2	PIP2;2_F	aaagaaaggccttcgctgga	00001d005421
	PIP2;2_R	tctccccacgctagatcgat	
ZmPIP2;3	PIP2;3_F	tgttggcccctcttccgattg	00001d051174
	PIP2;3_R	ccattggtcgtcccaggc	
ZmPIP2;4	PIP2;4_F	gttccggtgctagctcctctc	00001d017288
	PIP2;4_R	caggccttgtccttgttgtaga	
ZmPIP2;5	PIP2;5_F	ctccttcagccgctagatcg	00001d003006
	PIP2;5_R	ctgtccttgtccagcctctg	
ZmPIP2;6	PIP2;6_F	tcaggtcaagtgttgcagct	00001d019565
	PIP2;6_R	cacacttgctttccacaccg	
FPGS	FPGS_F	atctcgttggggatgtcttg	00001d048514
	FPGS_R	agcaccgttcaaatgtctcc	
MEP	MEP_F	tgtactcggcaatgctcttg	00001d018359
	MEP_R	tttgatgctccaggcttacc	
